# An Analytical Study of Electromagnetic Deep Penetration Conditions and Implications in Lossy Media through Inhomogeneous Waves

**DOI:** 10.3390/ma11091595

**Published:** 2018-09-03

**Authors:** Paolo Baccarelli, Fabrizio Frezza, Patrizio Simeoni, Nicola Tedeschi

**Affiliations:** 1 Department of Engineering, Roma Tre University, 00146 Rome, Italy; paolo.baccarelli@uniroma3.it; 2 Department of Information Engineering, Electronics and Telecommunications (DIET), Sapienza University of Rome, 00184 Rome, Italy; nicola.tedeschi@uniroma1.it; 3 National Transport Authority (NTA), Harcourt Lane, D02WT20 Dublin, Ireland; patrizio.simeoni@nationaltransport.ie

**Keywords:** lossy media, deep penetration, electromagnetic propagation in absorbing media, inhomogeneous waves, leaky waves

## Abstract

This paper illustrates how the penetration of electromagnetic waves in lossy media strongly depends on the waveform and not only on the media involved. In particular, the so-called inhomogeneous plane waves are compared against homogeneous plane waves illustrating how the first ones can generate deep penetration effects. Moreover, the paper provides examples showing how such waves may be practically generated. The approach taken here is analytical and it concentrates on the deep penetration conditions obtained by means of incident inhomogeneous plane waves incoming from a lossless medium and impinging on a lossy medium. Both conditions and constraints that the waveforms need to possess to achieve deep penetration are analysed. Some results are finally validated through numerical computations. The theory presented here is of interest in view of a practical implementation of the deep penetration effect.

## 1. Introduction

The achievement of electromagnetic deep penetration is of extreme importance in many applications, e.g., detection of buried or immersed objects, information transmission in lossy media, material analysis and microscopy, and interaction with biological tissues. A variety of techniques are commonly employed to improve the penetration, depending on the field of application, which, in turn, is strongly dependent on both the frequencies and the media involved. Typical examples employed in the literature span from antenna optimisation techniques [1,2] to the appropriate use of coupling liquids [3]; sometimes, the two approaches are combined, e.g., the case reported in [4] where the bolus medium is also chosen as dielectric for the antenna to improve the performances. Often, to improve the penetration, it is necessary to reduce the frequency, and this comes with undesired effects such as loss of resolution in imaging applications.

An alternative approach may consist of improving penetration by designing structures able to generate inhomogeneous plane waves with specific properties that will be discussed here. This approach could lead to an increase in penetration without reducing frequency, and, consequently, resolution [5,6]. Based on the preliminary results presented in [7], the analysis performed in this paper provides a complete description of the deep penetration phenomenon by means of inhomogeneous plane waves at the planar boundary between a lossless medium and a lossy one. The paper starts with a theoretical review of the physical problem, and, then, in the results section, new findings are illustrated: a unique identification of the inhomogeneous-wave solutions which can exhibit deep penetration is provided in the first subsection, distinguishing them from the ones that can only experience attenuation in lossy media. In the second subsection, a specific attention is given to the determination of the requisites that media have to possess in order to allow deep penetration solutions, combined with the properties that the incident wave has to exhibit in terms of the amplitudes of the attenuation and phase vectors. Finally, conditions for deeper penetration, i.e., negative normal component of the transmitted attenuation vector in the lossy medium, will be discussed in the final subsection. A brief discussion on the results found follows. The objective of this study is to provide the complete theoretical and analytical details needed to drive the experimental verification of this important phenomenon and, possibly, to design practical antennas, for instance Leaky-Wave Antennas (LWAs) [8], capable of generating the suitable field distributions.

## 2. Theoretical Background

### 2.1. Problem Definition

**Figure 1:**
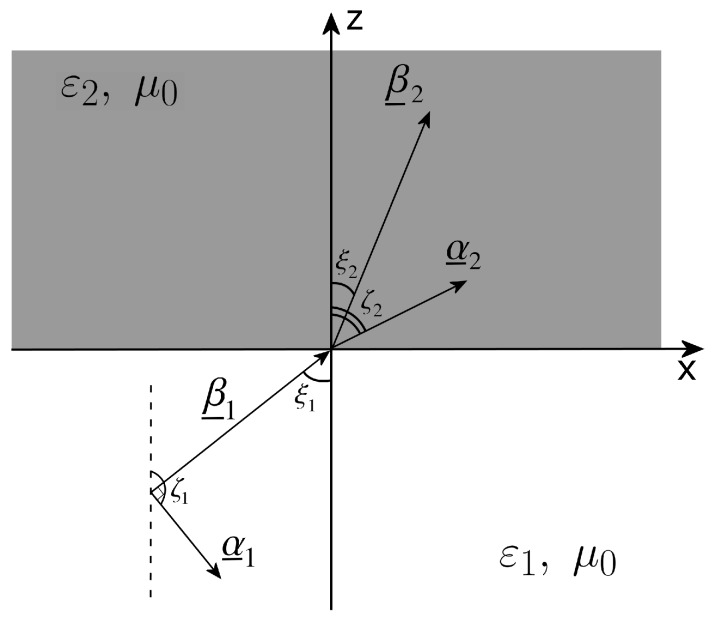
Geometry of the problem.

The problem analyzed in this paper is illustrated in Figure 1, where the incidence of an inhomogeneous plane wave on a plane separation surface between two media is considered. (The xz plane is heretaken, without loss of generality). The incident wave is incoming from medium 1 and impinges on the separation surface producing, in general, a reflected wave in medium 1 (not shown in Figure 1) and a transmitted wave in medium 2.

In Figure 1, we suppose both media non-magnetic, homogeneous and isotropic. The medium 1 is considered lossless, with a real relative permittivity $\varepsilon_{1}$, while the medium 2 dissipative, with a complex relative permittivity $\varepsilon_{2}=\varepsilon_{2}^{\prime }-j\varepsilon_{2}^{\prime \prime }=\varepsilon_{2}^{\prime }-j\sigma_{2}/\left(\varepsilon_{0}\omega \right)$, where *j* is the imaginary unit, $\varepsilon_{2}^{\prime }$ and $\varepsilon_{2}^{\prime \prime }$ are the real and imaginary part of the complex relative permittivity, respectively, $\varepsilon_{0}$ is the vacuum (absolute) permittivity and $\sigma_{2}$ is the electric conductivity. The (absolute) magnetic permeability of both media is indicated with $\mu_{0}$ and corresponds to the one of a vacuum.

All plane waves considered are of the form $\exp\left[-j\left({\underline{k}}_{i}\cdot {\underline{r}}_{i}-\omega t\right)\right]$, where i=1,2, ${\underline{r}}_{i}$ is the vector position, and ${\underline{k}}_{i}$ is the complex wave vector. The wave vector of the incident wave is expressed as ${\underline{k}}_{1}={\underline{\beta }}_{1}-j{\underline{\alpha }}_{1}$, where ${\underline{\beta }}_{1}$ is the phase vector and ${\underline{\alpha }}_{1}$ is the attenuation vector: in this case, it must be ${\underline{\beta }}_{1}⊥{\underline{\alpha }}_{1}$, the medium 1 being lossless [9]. Analogously, the wave vector of the medium 2 is defined as ${\underline{k}}_{2}={\underline{\beta }}_{2}-j{\underline{\alpha }}_{2}$, with ${\underline{\beta }}_{2}$ and ${\underline{\alpha }}_{2}$ the relevant phase and attenuation vectors and ${\underline{\beta }}_{2}\;/\;⊥\;{\underline{\alpha }}_{2}$, the medium 2 being lossy. $k_{1}=k_{0}\sqrt{\varepsilon_{1}}$ and $k_{2}=k_{0}\sqrt{\varepsilon_{2}}$ are the wavenumbers of the incident and transmitted wave, respectively, with $k_{0}$ the free-space wavenumber; $\xi_{1}$ and $\zeta_{1}$ indicate the angles that ${\underline{\beta }}_{1}$ and ${\underline{\alpha }}_{1}$, respectively, form with the normal to the separation surface, and $\xi_{2}$ and $\zeta_{2}$ the equivalent relevant angles for ${\underline{\beta }}_{2}$ and ${\underline{\alpha }}_{2}$ (see, again, Figure 1). The transmitted wave vector in the lossy medium is always complex, while $\zeta_{2}$ and $\xi_{2}$ are real, as follows from the Adler–Chu–Fano formalism [10].

### 2.2. The Deep Penetration Condition

In the lossy medium 2, defining $\eta_{2}=\left|\zeta_{2}-\xi_{2}\right|<90^{\circ }$ as the angle formed by ${\underline{\beta }}_{2}$ and ${\underline{\alpha }}_{2}$, it is possible to represent the amplitude $\beta_{2}$ of the phase vector ${\underline{\beta }}_{2}$ following Equation (8.14a) of [10], that, when non-magnetic media are considered, becomes:

(1) $$\beta_{2}=\frac{k_{0}\sqrt{\varepsilon_{2}^{\prime }}}{\sqrt{2}}\sqrt{\sqrt{1+{\left(\frac{\varepsilon_{2}^{\prime \prime }}{\varepsilon_{2}^{\prime }\cos\eta_{2}}\right)}^{2}}+1}$$

The amplitude $\alpha_{2}$ of ${\underline{\alpha }}_{2}$ is instead described by using Equation (8.14b) in [10] (The right-hand side of Equation (2) follows from Equations (8) and (9) of [10]), which, in the case of non-magnetic media, becomes:

(2) $$\begin{array}{c} \alpha_{2}=\frac{k_{0}\sqrt{\varepsilon_{2}^{\prime }}}{\sqrt{2}}\sqrt{\sqrt{1+{\left(\frac{\varepsilon_{2}^{\prime \prime }}{\varepsilon_{2}^{\prime }\cos\eta_{2}}\right)}^{2}}-1}\\ =\frac{\frac{\sigma_{2}}{2}\sqrt{\frac{\mu_{0}}{\varepsilon_{0}}}\sqrt{\frac{1}{\varepsilon_{2}^{\prime }}}}{\sqrt{\sqrt{1+{\left(\frac{\varepsilon_{2}^{\prime \prime }}{\varepsilon_{2}^{\prime }\cos\eta_{2}}\right)}^{2}}+1}}<\beta_{2} \end{array}$$

The penetration depth is commonly defined as the distance, inside a lossy medium, at which the electric field, or the magnetic field, amplitude reduces of a factor 1/e with respect to its maximum value at the separation interface. Typically, in the literature, this distance is assumed as $\delta =1/\alpha_{2}$, but, clearly, in directions different from the one of the attenuation vector, the penetration assumes different values. Here, we are interested in the attenuation in the direction orthogonal to the separation surface between lossless and lossy media and we assume that the transmitted attenuation vector is not, in general, orthogonal to the interface, i.e., $\zeta_{2}\neq 0^{\circ }$. As a consequence, the penetration can be defined as $\delta =1/(\alpha_{2}\cos\zeta_{2})$. Therefore, the penetration is larger than the one obtained when the incident wave is homogeneous, i.e., when $\zeta_{2}=0^{\circ }$.

**Figure 2:**
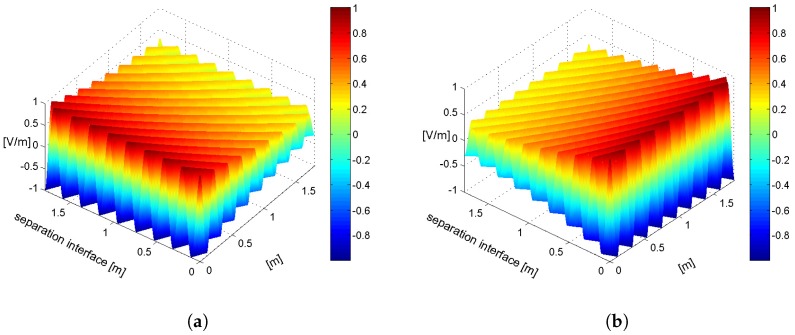
Plane wave of 1 V amplitude and 1 GHz frequency, propagating in a lossy medium with permittivity $\varepsilon =4\varepsilon_{0}$, permeability $\mu =\mu_{0}$, and σ=0.005S/m. A propagation angle of $45^{\circ }$ with respect to the plane separation surface is considered here. In both figures, the vertical axis represents the field amplitude, expressed in V/m, while the other two axes represent the separation surface [m], and the direction orthogonal to the separation surface [m], respectively. The vertical gradient bar, on the right-hand side of both figures, associates different colors to different amplitudes of the electric field. (**a**) wave in a lossy medium: attenuation vector normal to the separation surface; (**b**) wave in a lossy medium: attenuation vector parallel to the separation surface.

It is well known that, if $\alpha_{1}=0$, then it must be $\zeta_{2}=0^{\circ }$, as a direct consequence of the conservation of the tangential component of the fields [9] (independently from the incidence angle). A typical example of a plane wave in a lossy medium attenuating normally to the separation surface is shown in Figure 2a, where Equations (1) and (2) are described through Matlab [11] (MathWorks, Version R2011b) for the case $\xi_{2}=45^{\circ }$ and $\zeta_{2}=0^{\circ }$. If, instead, an inhomogeneous plane wave impinges on the separation surface between lossless and lossy media, then there exists a component of ${\underline{\alpha }}_{1}$ tangent to the separation surface, and therefore $\zeta_{2}\neq 0^{\circ }$. A specific example is shown in Figure 2b, where again Equations (1) and (2) are plotted using Matlab, but this time representing a wave in a lossy medium propagating with $\xi_{2}=45^{\circ }$ and $\eta_{2}=45^{\circ }$, which implies $\zeta_{2}=90^{\circ }$. The latter result illustrates the attracting scenario of an electromagnetic field which penetrates with no decay within the lossy medium, since there is no component of the attenuation vector along the direction normal to the separation surface and opposing to penetration, ${\underline{\alpha }}_{2}$ being directed along the *x*-axis.

The incidence from a lossless medium has been treated analytically in [7], finding the condition that allows for the attenuation vector of the transmitted wave inside the lossy medium to be parallel to the separation surface. In particular, in [7], the specific condition $\zeta_{2}=90^{\circ }$ is treated; such a condition implies a solution for the transmitted wave equivalent to the one shown in Figure 2b. When the medium 1 is lossless, $\zeta_{2}=90^{\circ }$ is met if the amplitude of the incident phase vector ${\underline{\beta }}_{1}$ is:

(3) β1≥β1c=k121+1+2Imk22k122

As a consequence, the amplitude $\alpha_{1}$ of the incident attenuation vector ${\underline{\alpha }}_{1}$ is determined, the medium 1 being known as $\alpha_{1}=\sqrt{\beta_{1}^{2}-k_{1}^{2}}$.

The critical incident angle $\xi_{1}=\xi_{c}$ for which Equation (3) is verified is found to be the solution of the following Equation

(4) β1α1sin(2ξc)=Im(k22)

that, according to the authors in [7], is given by:

(5) $$\xi_{1}=\xi_{c}=\frac{1}{2}\gamma ,\;\;\;\;\mathrm{with}\;\;0<\xi_{c}\leq \frac{\pi }{4}$$

where γ=arcsinIm(k22)/(β1α1). The larger the $\beta_{1}$ is, the smaller is the critical angle $\xi_{c}$ for which $\zeta_{2}=90^{\circ }$.

In this paper, the deep penetration phenomenon envisioned in [7] will be fully described, by suitably extending the theory and providing all the needed analytical details.

## 3. Results

### 3.1. The Direction of the Attenuation Vector in the Lossless Medium and Its Physical Consequences

**Figure 3:**
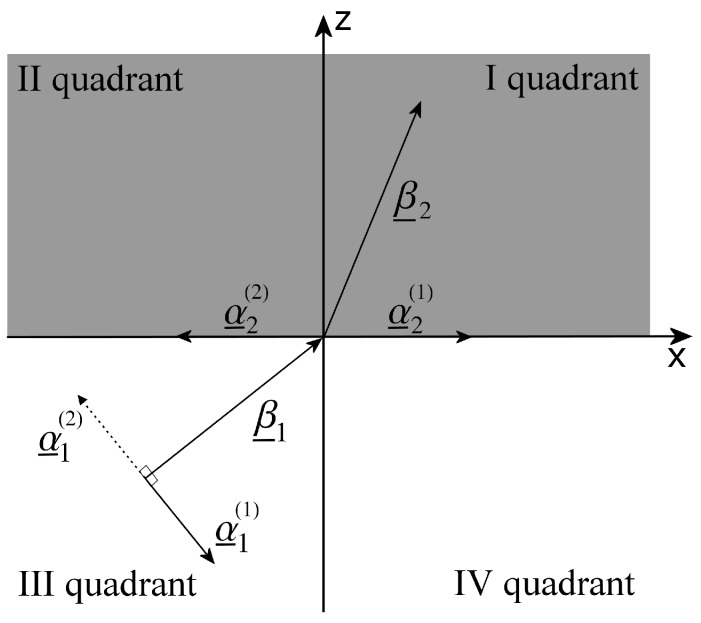
Proper and improper inhomogeneous waves at the interface between a lossless medium and a lossy one.

In [7], it is simply assumed that the deep penetration phenomenon can be obtained when ${\underline{\beta }}_{1}$ and ${\underline{\alpha }}_{1}$ are orthogonal, but no hypothesis is provided around the direction of the vector ${\underline{\alpha }}_{1}$, which theoretically can form with ${\underline{\beta }}_{1}$ an angle equal to $\pm 90^{\circ }$, as shown in Figure 3. Here, the discussion on the impact of the direction of the attenuation vector ${\underline{\alpha }}_{1}$ and the relevant practical implications on how to obtain the deep penetration will be studied in detail.

The ambiguity on the sign of the angle formed by ${\underline{\beta }}_{1}$ and ${\underline{\alpha }}_{1}$ corresponds to an ambiguity in the direction of the attenuation vector, as shown in Figure 3. We are going to demonstrate that only one direction of the attenuation vector of the incident wave allows for deep penetration, and specifically the one corresponding to the angle $+90^{\circ }$ (see ${\underline{\alpha }}_{1}^{\left(1\right)}$ in Figure 3); the other solution, i.e., relevant to the angle $-90^{\circ }$ (see ${\underline{\alpha }}_{1}^{\left(2\right)}$ in Figure 3), produces, instead, an attenuated transmitted wave.

Since the incident inhomogeneous wave can admit ${\underline{\alpha }}_{1}={\underline{\alpha }}_{1}^{\left(1\right)}$ and ${\underline{\alpha }}_{1}={\underline{\alpha }}_{1}^{\left(2\right)}=-{\underline{\alpha }}_{1}^{\left(1\right)}$, the condition for deep penetration $\zeta_{2}=\pm 90^{\circ }$ translates either into ${\underline{\alpha }}_{2}={\underline{\alpha }}_{2}^{\left(1\right)}$ or into ${\underline{\alpha }}_{2}={\underline{\alpha }}_{2}^{\left(2\right)}$, respectively, as is shown in Figure 3. With reference to Figure 3, the phase vector ${\underline{\beta }}_{1}$ is incoming from the III quadrant and impinges on the origin of the axes: it follows that the vector ${\underline{\beta }}_{2}$ can only be in the I quadrant due to the conservation of the tangential component of ${\underline{\beta }}_{1}$. In the case illustrated, the angle formed by ${\underline{\alpha }}_{2}$ and ${\underline{\beta }}_{2}$ must be always less than $90^{\circ }$ (it is ${\underline{\beta }}_{2}\cdot {\underline{\alpha }}_{2}=\omega^{2}\mu \varepsilon_{2}^{\prime \prime }\varepsilon_{0}/2>0$), therefore ${\underline{\alpha }}_{2}^{\left(2\right)}$ can never be a solution of our problem, the only valid solution is represented by ${\underline{\alpha }}_{2}^{\left(1\right)}$. As a consequence, ${\underline{\alpha }}_{1}^{\left(2\right)}$ can never allow for deep penetration, but it can only admit an attenuated solution with ${\underline{\alpha }}_{2}$ laying in the second quadrant (again for the conservation of tangential component); on the opposite, the solution represented by ${\underline{\alpha }}_{1}^{\left(1\right)}$ guarantees, as predicted, a deep penetration effect, allowing ${\underline{\alpha }}_{2}^{\left(1\right)}$ as a valid solution.

We note that the inhomogeneous wave characterized by ${\underline{\alpha }}_{1}^{\left(1\right)}$ in the III quadrant of Figure 3 corresponds to a field that grows in the direction normal to the separation surface (entering medium 2), while the ${\underline{\alpha }}_{1}^{\left(2\right)}$ one to a decreasing field. Inhomogeneous waves of the former kind are also known as improper leaky waves, while those of the latter kind as proper leaky waves [8]. Hence, we conclude that the deep penetration phenomenon can only be obtained if the impinging inhomogenous wave in the lossless medium is an improper leaky wave.

**Figure 4:**
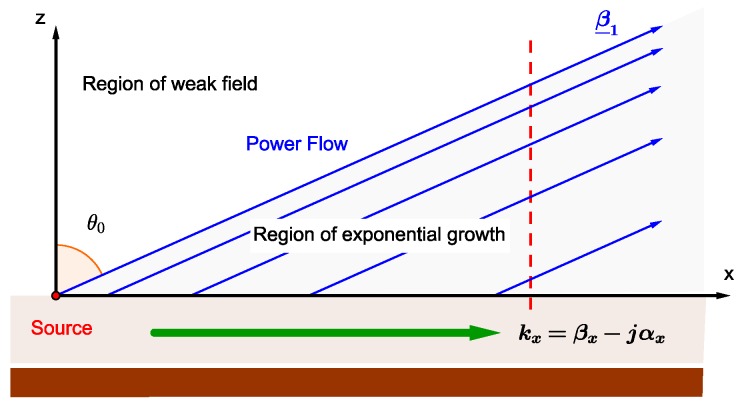
Ray pictures for the physical interpretation of an improper leaky-wave field.

As is known, an improper leaky wave violates the Sommerfeld condition [9], but, even though this condition is violated, such a wave can be produced by a finite source in a limited region of space. In Figure 4, a simple ray picture of an improper leaky wave launched by a source at x=0 along the positive *x*-direction of an open waveguide is shown. The field on the aperture (z=0) has the form of a leaky wave, e.g., the *y*-component of the electric field is $E_{y}\left(x,0\right)=A$ exp$\left(-jk_{x}x\right)$, where the complex wavenumber of the leaky wave is given by $k_{x}=\beta_{x}-j\alpha_{x}$, with $\beta_{x}$ and $\alpha_{x}$ the relevant phase and attenuation constant, respectively [8]. The wave decays exponentially for x→+∞; however, the leaky-wave field may be dominant on the aperture out to a considerable distance from the source. In addition, the exact field due to the leaky-wave aperture distribution may be calculated by using a simple Fourier transform approach in terms of an appropriate plane-wave expansion [8]. This interpretation is coherent with [12], where it is demonstrated that the radiation generated can be interpreted as the sum of two components: a leaky wave and a space wave, of which the first one is more relevant close to the source while the other prevails at a large distance from the source. If the leaky wave is excited strongly, its power is transferred to the space wave and carried to the far field, and the radiation peak is obtained at an angle close to the propagation angle of that complex wave.

Rays, in Figure 4, indicate the direction of the power flow in the air region, which for an inhomogeneous plane-wave field is in the direction of the phase vector ${\underline{\beta }}_{1}$, i.e., at an angle $\theta_{0}=\tan^{-1}\left(\beta_{1x}/\beta_{1z}\right)$ with respect to the *z*-axis. The separation between the rays indicates the strength of the field. In particular, closer separation corresponds to a stronger field. A leakage “shadow boundary” exists at the angle $\theta_{0}$ from the *z*-axis that separates a “wedge-shaped region”, i.e., $\theta_{0}<\theta <90^{\circ }$, where the field is similar to that of an inhomogeneous plane wave (improper leaky wave), from the region defined by $0^{\circ }<\theta <\theta_{0}$, where the field is very weak. In practice, as an observer moves vertically away from the aperture, e.g., along the red dashed line in Figure 4, the field level will increase exponentially up to the leakage shadow boundary, and will then decrease very quickly above this boundary. Therefore, the field from this leaky wave will not increase indefinitely in the vertical direction, and will not violate the radiation condition at infinity [12,13]. Hence, if the lossy medium is placed in the wedge-shaped region highlighted in Figure 4, improved penetration can suitably occur. Hence, the inhomogeneous wave is a model valid to represent physically achievable waves, as the improper leaky waves in the near field of a LWA, only in a well-defined region of the space that, in particular, can include the lossy medium, thus allowing for deep penetration.

In conclusion, a monodimensional planar LWA, operating in the improper leaky-wave regime [8] and posed in medium 1 at a given distance from and on a plane parallel to the separation surface with the dissipative medium 2, can be suitably used to obtain deep penetration of electromagnetic field in lossy media, provided that the lossy volume is positioned in the near-field wedge-shaped region of the LWA.

LWAs promise a deep penetration effect in the near field, therefore they are suitable in all scenarios in which the penetration needs to be sustained for a limited number of wavelengths, as, for instance, it was illustrated in [5,6], where LWAs were found to guarantee higher penetration in biomedical applications such as hyperthermia.

### 3.2. Complete Set of Conditions for Deep Penetration

The complete set of solutions for Equation (4) in the interval $\xi_{1}\in \left[0,90^{\circ }\right]$ is given by:

(6) $$\begin{array}{c} \xi_{c1}=\frac{1}{2}\gamma \end{array}$$

(7) $$\begin{array}{c} \xi_{c2}=\frac{\pi }{2}-\frac{1}{2}\gamma \end{array}$$

Equation (6) being the one reported in [7]. In this section, both the expressions of $\xi_{c}$ in Equations (6) and (7) will be physically interpreted and it will be shown that the two obtained solutions may not always imply deep penetration. An accurate analysis of the two media involved will follow, in order to determine when deep penetration is practically achievable.

The first medium is supposed lossless, therefore if we consider the incident inhomogeneous wave suitable for obtaining deep penetration (i.e., see ${\underline{\alpha }}_{1}^{\left(1\right)}$ in Figure 3) and we apply the generalized Snell laws and the separability condition in the second lossy medium, we obtain the following Equations:

(8) $$\begin{array}{c} \beta_{1}\sin\xi_{1}=\beta_{2}\sin\xi_{2} \end{array}$$

(9) $$\begin{array}{c} \alpha_{1}\sin\zeta_{1}=\alpha_{2}\sin\zeta_{2} \end{array}$$

(10) β22−α22=Re(k22)

(11) 2β2α2cos(ζ2−ξ2)=Im(k22)

Equation (9) can be rewritten exploiting the orthogonality of the phase and attenuation vectors in the first medium. Since the angle formed by ${\underline{\beta }}_{1}$ and ${\underline{\alpha }}_{1}$ is $+90^{\circ }$, from Figure 1, it follows that:

(12) $$\begin{array}{cc} \zeta_{1}=\frac{\pi }{2}+\xi_{1}\to \alpha_{1}\sin\zeta_{1} & =\alpha_{1}\sin\left(\frac{\pi }{2}+\xi_{1}\right)\\ & =\alpha_{1}\cos\xi_{1} \end{array}$$

Hence, by using (9) and (12), it is:

(13) $$\begin{array}{c} \alpha_{1}\cos\xi_{1}=\alpha_{2}\sin\zeta_{2} \end{array}$$

and, introducing (8) and (13) into (11), we obtain:

(14) 2β2α2cosζ2cosξ2+α1β1sin2ξ1=Im(k22)

If we look for the critical angle at which the transmitted attenuation vector ${\underline{\alpha }}_{2}$ is parallel to the interface, we must impose $\zeta_{2}=90^{\circ }$ in (14), obtaining (4). However, if we look for the critical angle at which the transmitted phase vector ${\underline{\beta }}_{2}$ is parallel to the interface, we must impose $\xi_{2}=90^{\circ }$ obtaining, again, (4). The conditions found classify two very different physical problems, the former represents deep penetration, while the latter reminds one very closely of the so-called Zenneck wave at the interface between two lossy media [14], but it differs from the Zenneck wave for the absence of the total-transmission effect [15]. Here, we need to distinguish the two physical problems, i.e., we must find a way to understand when a transmitted phase or attenuation vector parallel to the interface can be obtained, respectively. To ascertain such a condition, we can consider the expressions of the magnitudes of the transmitted phase and attenuation vectors in medium 2 given in [16]

(15) β2=|k∥|2+Re(k22)+|k∥2−k22|2

(16) α2=|k∥|2−Re(k22)+|k∥2−k22|2

where $k_{∥}$ indicates the component of ${\underline{k}}_{1}$ parallel to the separation interface.

Let us consider the absolute value under square root: hence, by using (4), i.e., under the hypothesis that $\zeta_{2}=90^{\circ }$ or $\xi_{2}=90^{\circ }$, we can find the following expression:

(17) |k∥2−k22|=|β12sin2ξ1−α12cos2ξ1−Re(k22)|=|Re(k∥2)−Re(k22)|

Substituting (17) in (15) and (16), we found that the solution $\xi_{2}=90^{\circ }$, corresponding to the transmitted phase vector parallel to the interface, is consistent with Re(k∥2)≥Re(k22), while $\zeta_{2}=90^{\circ }$, corresponding to the transmitted attenuation vector parallel to the interface, requires Re(k∥2)<Re(k22).

It is now important to understand when the two solutions (6) and (7) are relevant either to the case $\xi_{2}=90^{\circ }$ or $\zeta_{2}=90^{\circ }$. For the sake of brevity, we will call the solution of the “phase” type when it is relevant to the case $\xi_{2}=90^{\circ }$ and of the “attenuation” type when it is relevant to the case $\zeta_{2}=90^{\circ }$ (i.e., the deep penetration case).

Let us impose the phase type solution, Re(k∥2)−Re(k22)≥0 in (17), it follows β12sin2ξ1−α12cos2ξ1−Re(k22)≥0. Then, by applying bisection-trigonometrical formulas, it is:

(18) cos2ξ1≤−2Re(k22)−k12β12+α12

Furthermore, defining the quantity Ψ as follows:

(19) $$\begin{array}{c} \Psi =\frac{k_{1}^{2}}{\beta_{1}^{2}+\alpha_{1}^{2}}\left(2\frac{\varepsilon_{2}^{\prime }}{\varepsilon_{1}}-1\right) \end{array}$$

Equation (18) can be written as:

(20) $$\begin{array}{c} \cos\left(2\xi_{1}\right)\leq -\Psi \end{array}$$

Using the two conditions of Equations (6) and (7), (20) can be written in terms of γ, so $\xi_{c1}$ is of the phase type when cosγ≤−Ψ, otherwise it is of the attenuation type; $\xi_{c2}$ is of the phase type if cosγ≥Ψ, otherwise it is of the attenuation type. From such conditions, it is possible to foresee the type of the solutions in many cases, in fact (recalling that $\gamma \in [0,90^{\circ }]$): if Ψ<−1, both $\xi_{c1}$ and $\xi_{c2}$ solutions are of the phase type; if −1≤Ψ<0, the $\xi_{c1}$ type is determined by the value of γ, while $\xi_{c2}$ is of the phase type; if Ψ=0, $\xi_{c1}$ is of the attenuation type, while $\xi_{c2}$ is of the phase type; if 0<Ψ≤1, $\xi_{c1}$ is of the attenuation type, while the $\xi_{c2}$ type is determined by the value of γ; finally, if Ψ>1, both the solutions are of the attenuation type.

**Figure 5:**
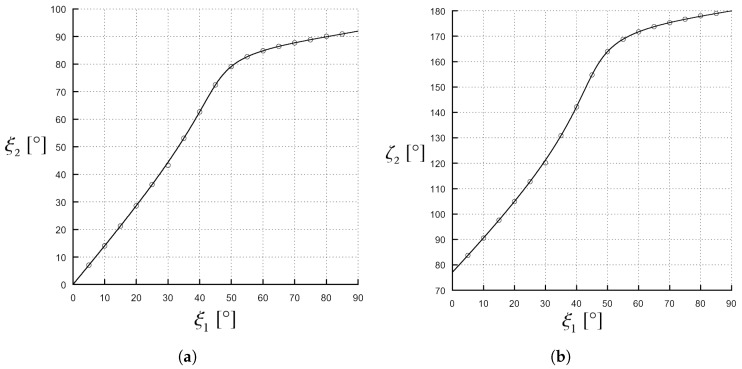
Values of $\zeta_{2}$ and $\xi_{2}$ angles in the case of $\varepsilon_{1}=2$, $\varepsilon_{2}^{\prime }=1$, $\varepsilon_{2}^{\prime \prime }=0.1$ and $\beta_{1}=1.01\beta_{1c}$. The angles have been computed by (solid line) a numerical code, implementing the analytical expressions, and by (circles) full-wave simulations on a commercial software. (**a**) phase solution, the incidence angle needs to be clearly larger than $45^{\circ }$, the propagation vector of the transmitted wave is parallel to the separation surface for an incidence angle of $80^{\circ }$; (**b**) attenuation solution, the incidence angle needs to be clearly smaller than $45^{\circ }$, the attenuation vector of the transmitted wave is parallel to the separation surface for an incidence angle $\xi_{1}\approx 15^{\circ }$. (Note that increasing the value of the $\xi_{1}$ angle, the value of $\zeta_{2}$ becomes larger than $90^{\circ }$.)

**Figure 6:**
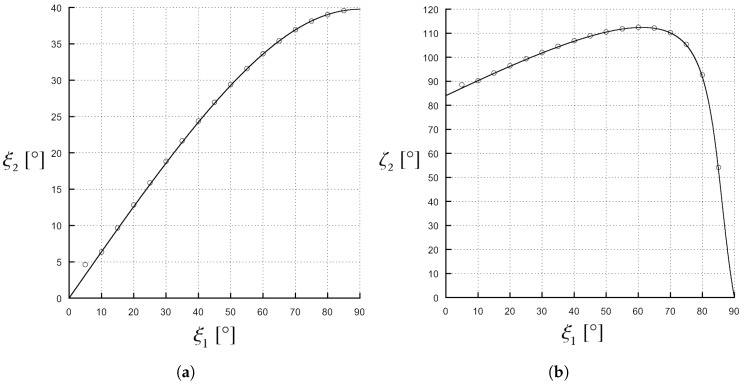
Values of $\zeta_{2}$ and $\xi_{2}$ angles in the case of $\varepsilon_{1}=2$, $\varepsilon_{2}^{\prime }=5$, $\varepsilon_{2}^{\prime \prime }=0.1$ and $\beta_{1}=1.01\beta_{1c}$. The angles have been computed by (solid line) a numerical code, implementing the analytical expressions, and by (circles) full-wave simulations on a commercial software. (**a**) $\xi_{2}$ as a function of $\xi_{1}$ is shown here. A phase solution is never possible, the $\xi_{2}$ angle is acute for each $\xi_{1}$ value; (**b**) $\zeta_{2}$ as a function of $\xi_{1}$ is shown here. There are two attenuation solutions, one for $\xi_{1}\approx 10^{\circ }$ and the other for $\xi_{1}\approx 80^{\circ }$; in particular, one solution is found for $\xi_{1}<45^{\circ }$ and the other for $\xi_{1}>45^{\circ }$.

Important physical constraints for the media involved in the deep penetration phenomenon can be derived by the previous analysis. Looking at the expression (19), we see that the sign of Ψ is determined by the ratio of the real parts of the permittivities (note that both medium 1 and medium 2 are assumed non-magnetic). The case in which both the solutions are of the phase type (i.e., Ψ<−1) requires $\varepsilon_{2}^{\prime }<0$: this case, which is not uncommon at the optic frequencies (e.g., gold and silver exhibit $\varepsilon_{2}^{\prime }<0$ values at such frequencies), is not probable at microwave frequencies; therefore, we can say that, in the case of microwave radiation, adopted in many applications, a deep penetration solution always exists, while for different frequency ranges may not be guaranteed. Furthermore, we observe that Ψ≥0 when $2\varepsilon_{2}^{\prime }\geq \varepsilon_{1}$. In this scenario, that is typically met, e.g., the case of incidence from a vacuum, the solution $\xi_{c1}$ is always of the attenuation type. The behavior of $\xi_{c2}$ depends on the characteristics of the incident wave and in particular on the parameter $\beta_{1}/k_{1}$: in fact, the quantity in brackets in (19) is multiplied by a function of such parameter that is a decreasing function bounded in the interval (0,1). As a consequence, the larger $\beta_{1}/k_{1}$ is, the smaller Ψ is, making the determination of the type of $\xi_{c2}$ dependent on γ even for a high $\varepsilon_{2}^{\prime }/\varepsilon_{1}$ ratio. Numerical results are shown in Figure 5 and Figure 6 for the cases Ψ=0 and Ψ>1, respectively. The angles have been computed both with a numerical code in Matlab, implementing the analytical expressions of $\xi_{2}$ and $\zeta_{2}$ as a function of $\xi_{1}$ obtained from Equations (8) and (9), and with full-wave simulations on Comsol Multiphysics [17] (COMSOL Inc., Version 5.3 ), a commercial software based on the Finite-Element Method. In Figure 5, a medium 1, with $\varepsilon_{1}=2$, and a medium 2, with $\varepsilon_{2}^{\prime }=1$ and $\varepsilon_{2}^{\prime \prime }=0.1$, are considered, the phase vector of the incident wave is $\beta_{1}=1.01\beta_{1c}$. $\xi_{2}$ as a function of $\xi_{1}$ is illustrated in Figure 5a: the only solution of phase type appears when $\xi_{1}\approx 80^{\circ }$. In Figure 5b, $\zeta_{2}$ as a function of $\xi_{1}$ is shown: the only solution of attenuation type appears for $\xi_{1}\approx 15^{\circ }$ and after this value $\zeta_{2}$ keeps incrementing for higher $\xi_{1}$ values. As a consequence, we observe a wave with $\zeta_{2}>90^{\circ }$ and a negative normal component of the attenuation vector in medium 2. Finally, comparing Figure 5a with Figure 5b, it can be seen that $\xi_{2}$ grows when $\zeta_{2}$ also is increasing; this means that $\beta_{2}$ vector tends to be parallel to the separation surface when the penetration gets stronger: the combined effects of the two vectors should therefore be considered in practical applications. Moreover, in Figure 5a, we can note that, for an amplitude of $\xi_{1}$ larger than $80^{\circ }$, the transmitted phase vector angle, $\xi_{2}$, assumes values larger than $90^{\circ }$, i.e., the transmitted phase vector is directed backwards in the half-space of origin of the incident wave; it is important to emphasize that this behaviour does not mean that the energy flow follows such a direction: in fact, when an inhomogeneous wave in a dissipative medium is considered, the direction of the energy flow is not the one of the phase vector, as well explained in [10,18].

In Figure 6, a typical scenario is shown instead in which both solutions are of attenuation type, i.e., Ψ>1: in this case, it is $\varepsilon_{1}=2$, $\varepsilon_{2}^{\prime }=5$, $\varepsilon_{2}^{\prime \prime }=0.1$, and $\beta_{1}=1.01\beta_{c}$. It can be noticed that the transmitted phase vector is never parallel to the interface (see Figure 6a) while the transmitted attenuation vector is parallel to the separation interface for two values of the incidence angle: namely, $\xi_{1}\approx 10^{\circ }$ and $\xi_{1}\approx 80^{\circ }$ (see Figure 6b). Also in this second scenario, similarly to what occurs for Ψ=0 (see Figure 5), there is a region in which $\zeta_{2}$ assumes values larger than $90^{\circ }$.

### 3.3. Electromagnetic Penetration around the Deep Penetration Condition

The minimal condition that allows for deep penetration is guaranteed by the angle $\zeta_{2}=90^{\circ }$. A stronger penetration can be achieved if the condition:

(21) $$\zeta_{2}>90^{\circ }$$

is satisfied: this condition, as said, was already found in the case of incidence from lossy medium in [16], and it is also satisfied for some incident angles in Figure 5b and Figure 6b, when the first medium is lossless.

In the conventional scenario in which the propagating wave attenuates entering in the lossy medium ($\zeta_{2}<90^{\circ }$), the components typical to the separation surface of both ${\underline{\beta }}_{2}$ and ${\underline{\alpha }}_{2}$ have the same sign, while (21) shows a case in which those two components must present opposite signs: this means that the field increases while propagating in the lossy medium. We are going to demonstrate that the condition of (21) can be obtained by studying the case in which both the incident wave (and in particular its operating frequency $f_{0}$ and incident angle $\xi_{c}$) and medium 1 are maintained constant, while the lossy medium 2 is varied in its $\varepsilon_{2}^{\prime \prime }$ value. This in turn is equivalent to consider an inhomogeneous plane wave designed to impinge on a separation surface with a lossy medium 2 characterised by a relative permittivity $\varepsilon_{2c}=\varepsilon_{2}^{\prime }-j\varepsilon_{2c}^{\prime \prime }$, so that $\zeta_{2}$ assumes the critical value $\zeta_{2c}=90^{\circ }$; then, such a wave is applied, with the same incidence angle, to an interface with a lossy medium for which $\varepsilon_{2}^{\prime \prime }\neq \varepsilon_{2c}^{\prime \prime }$. We note that this scenario has practical applicability because it describes the common case in which an antenna design is performed and then the antenna is exposed to a medium which does not fully match the one for which the structure was optimised.

Let us now assume that we met the condition of (3) for $\varepsilon_{2}^{\prime \prime }=\varepsilon_{2c}^{\prime \prime }$, so that $\zeta_{2}$ = $\zeta_{2c}$. Let us now decrease the imaginary part $\varepsilon_{2}^{\prime \prime }$ of $\varepsilon_{2}$ to a positive value smaller than $\varepsilon_{2c}^{\prime \prime }$. This new value of the imaginary relative permittivity implies different amplitudes and directions for the transmitted phase and attenuation vectors; let us call with $\beta_{2}^{\prime }$ and $\alpha_{2}^{\prime }$ the magnitudes of such vectors and with $\xi_{2}^{\prime }$ and $\zeta_{2}^{\prime }$ the new angles formed by those vectors with the normal to the separation surface, respectively. Consequently, the new wave vector in medium 2 is indicated with ${\underline{k}}_{2}^{\prime }$ and the wave number with $k_{2}^{\prime }$. From (11), having imposed $\varepsilon_{2}^{\prime \prime }<\varepsilon_{2c}^{\prime \prime }$ (i.e., 0<Im(k2′)<Im(k2)), it follows that:

(22) $$\begin{array}{c} \beta_{2}\alpha_{2}\cos\left(\xi_{2}-\frac{\pi }{2}\right)>\beta_{2}^{\prime }\alpha_{2}^{\prime }\cos\left(\xi_{2}^{\prime }-\zeta_{2}^{\prime }\right) \end{array}$$

where (22) can be rewritten as follows:

(23) $$\begin{array}{c} \beta_{2}\alpha_{2}\sin\left(\xi_{2}\right)>\beta_{2}^{\prime }\alpha_{2}^{\prime }\cos\left(\xi_{2}^{\prime }-\zeta_{2}^{\prime }\right) \end{array}$$

We considered the hypothesis in which the same wave incoming from a lossless material was applied to two different media with the same incident angle; therefore, using (8) and (13), the following system of Equations is obtained:

(24) $$\left\{\begin{array}{c} \beta_{1}\sin\xi_{1}=\beta_{2}\sin\xi_{2}\\ \alpha_{1}\sin\zeta_{1}=\alpha_{2}\\ \beta_{1}\sin\xi_{1}=\beta_{2}^{\prime }\sin\xi_{2}^{\prime }\\ \alpha_{1}\sin\zeta_{1}=\alpha_{2}^{\prime }\sin\zeta_{2}^{\prime } \end{array}\right.$$

Removing $\beta_{1}$ and $\alpha_{1}$ from (24), we have:

(25) $$\left\{\begin{array}{c} \beta_{2}^{\prime }\sin\xi_{2}^{\prime }=\beta_{2}\sin\xi_{2}\\ \alpha_{2}^{\prime }\sin\zeta_{2}^{\prime }=\alpha_{2} \end{array}\right.$$

Now, $\beta_{2}$ and $\alpha_{2}$ in (23) can be eliminated, finally having:

$$\begin{array}{cc} \beta_{2}^{\prime }\sin\left(\xi_{2}^{\prime }\right) & \alpha_{2}^{\prime }\sin\left(\zeta_{2}^{\prime }\right)>\beta_{2}^{\prime }\alpha_{2}^{\prime }\cdot \\ & \left[\cos\left(\xi_{2}^{\prime }\right)\cos\left(\zeta_{2}^{\prime }\right)+\sin\left(\xi_{2}^{\prime }\right)\sin\left(\zeta_{2}^{\prime }\right)\right] \end{array}$$

Simplifying the expression above, the following is obtained:

(26) $$\cos\left(\xi_{2}^{\prime }\right)\cos\left(\zeta_{2}^{\prime }\right)<0$$

With reference to Figure 3, from (26), it follows that ${\underline{\beta }}_{2}^{\prime }$ and ${\underline{\alpha }}_{2}^{\prime }$ need to be positioned one on the I quadrant and the other on the IV quadrant, and in particular they cannot both be in theI quadrant; the latter would be the solution for an attenuated transmitted wave. Moreover, no assumption was made on $\xi_{2}$, apart from the deep penetration condition that forces ${\underline{\beta }}_{2}^{\prime }$ to be in the first quadrant, while it was posed $\zeta_{2}=\zeta_{2c}$. Hence, it follows that it must be $\zeta_{2}^{\prime }>\zeta_{2c}$ because the attenuation vector ${\underline{\alpha }}_{2}^{\prime }$ needs to be positioned to the right of ${\underline{\beta }}_{2}^{\prime }$, according to the previous discussion (see Figure 3), in order to allow for deep penetration.

Considering $\varepsilon_{2}^{\prime \prime }>\varepsilon_{2c}^{\prime \prime }$ would have caused, on the contrary, ${\underline{\alpha }}_{2}^{\prime }$ and ${\underline{\beta }}_{2}^{\prime }$ both in theI or both in the IV quadrant: in particular, the continuity of the solution would have requested $\zeta_{2}^{\prime }<\zeta_{2c}$, so both ${\underline{\alpha }}_{2}^{\prime }$ and ${\underline{\beta }}_{2}^{\prime }$ lie in the first quadrant. This solution corresponds to pure attenuation of the transmitted wave in medium 2. This theoretical expansion permits us to state that, finding a $\beta_{1c}$ value for which (3) is satisfied (i.e., $\zeta_{2c}=90^{\circ }$), the deep penetration effect stops occurring (i.e., $\zeta_{2}^{\prime }<\zeta_{2c}$) for higher $\varepsilon_{2}^{\prime \prime }$ values, while the wave penetrates in medium 2 with an increasing electric field module for lower $\varepsilon_{2}^{\prime \prime }$ values (i.e., $\zeta_{2}^{\prime }>\zeta_{2c}$), thus giving rise to a deeper penetration effect.

This could state e.g., that, in any case, a lossy medium will attenuate the exponential increase of the field intensity of an improper leaky wave to a certain degree. In case the deep penetration condition $\beta_{1}=\beta_{1c}$ is met precisely, the exponential increase is completely compensated and the resulting behaviour is a constant intensity. If the losses in the medium are larger than expected, the exponential increase is overcompensated resulting in an exponential decay. If the losses are lower, there is still an exponential increase but with a reduced rate.

**Figure 7:**
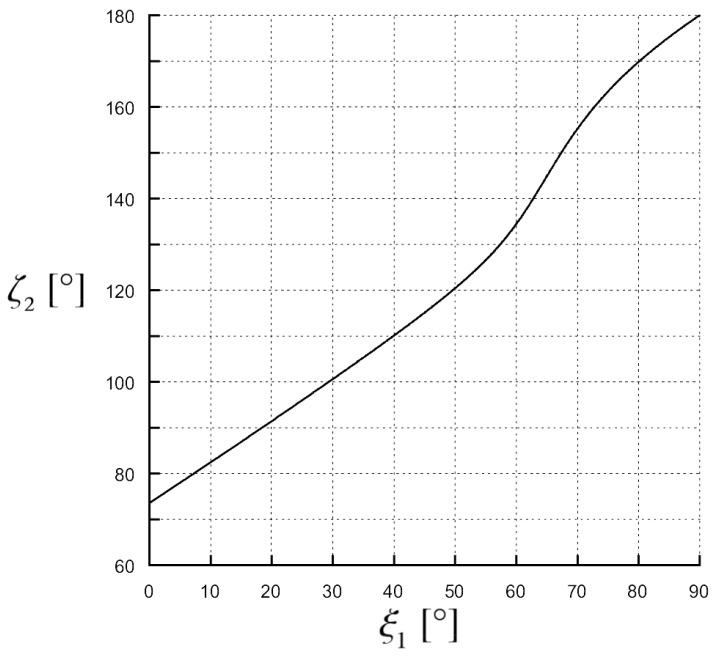
Transmitted angle of the attenuation vector, $\zeta_{2}$ in degrees, as a function of the incident angle of the phase vector, $\xi_{1}$ in degrees. Medium 1 is a vacuum, medium 2 has $\varepsilon_{2}^{\prime }=1$ and $\varepsilon_{2}^{\prime \prime }=0.5$. The magnitude of the incident phase vector is the critical one in Equation (3) when $\varepsilon_{2}^{\prime }=1$ and $\varepsilon_{2c}^{\prime \prime }=0.9$.

**Figure 8:**
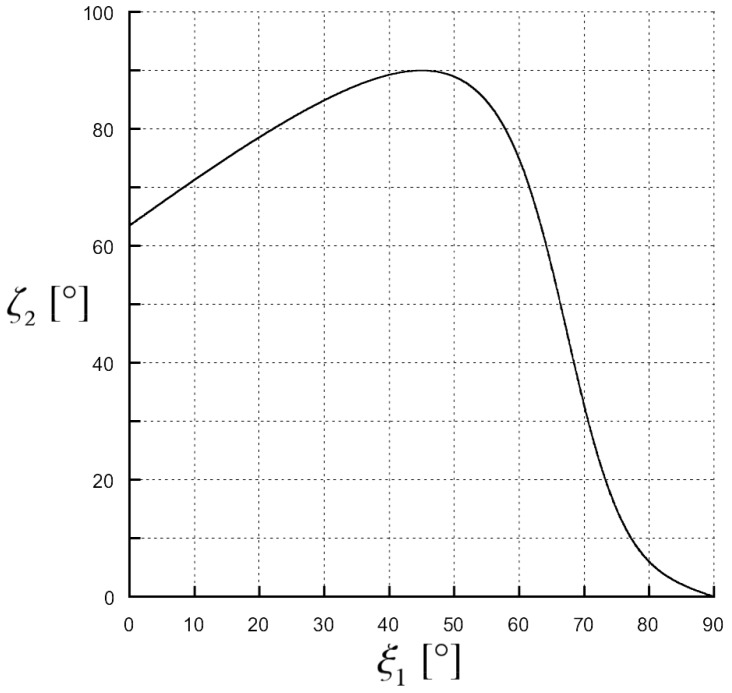
Transmitted angle of the attenuation vector, $\zeta_{2}$ in degrees, as a function of the incident angle of the phase vector, $\xi_{1}$ in degrees. Medium 1 is a vacuum, medium 2 has $\varepsilon_{2}^{\prime }=1$ and $\varepsilon_{2}^{\prime \prime }=0.9$. The magnitude of the incident phase vector is the critical one in Equation (3) when $\varepsilon_{2}^{\prime }=1$ and $\varepsilon_{2c}^{\prime \prime }=0.9$.

**Figure 9:**
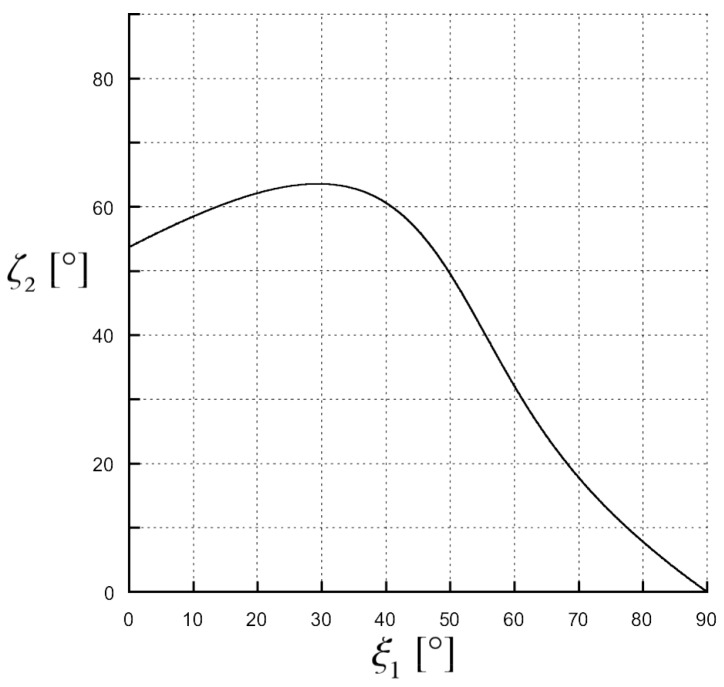
Transmitted angle of the attenuation vector, $\zeta_{2}$ in degrees, as a function of the incident angle of the phase vector, $\xi_{1}$ in degrees. Medium 1 is a vacuum, medium 2 has $\varepsilon_{2}^{\prime }=1$ and $\varepsilon_{2}^{\prime \prime }=1.4$. The magnitude of the incident phase vector is the critical one in Equation (3) when $\varepsilon_{2}^{\prime }=1$ and $\varepsilon_{2c}^{\prime \prime }=0.9$.

Numerical results, produced by a code in Matlab implementing the analytical expressions of $\xi_{2}$ and $\zeta_{2}$ as a function of $\xi_{1}$ obtained from Equations (8) and (9), are shown in Figure 7, Figure 8 and Figure 9 to prove the theory just explained. Let us assume a 1.55μm wavelength, the medium 1 is a vacuum, designing $\beta_{1}=\beta_{1c}$ to meet the condition $\zeta_{2c}=90^{\circ }$ when the second medium has $\varepsilon_{2}^{\prime }=1$ and $\varepsilon_{2c}^{\prime \prime }=0.9$ with an angle $\xi_{1c}=45^{\circ }$. In Figure 7, the transmitted attenuation angle in the case the second medium has $\varepsilon_{2}^{\prime \prime }=0.5<\varepsilon_{2c}^{\prime \prime }$ is shown. We can see that, in this case of increasing the incident angle, the transmitted attenuation angle reaches values greater than $90^{\circ }$, i.e., with reference to Figure 3, the transmitted attenuation vector lies in the IV quadrant. On the other hand, in Figure 8, the case $\varepsilon_{2}^{\prime \prime }=0.9=\varepsilon_{2c}^{\prime \prime }$ is considered. Here, we can see that the transmitted attenuation angle reaches the value $90^{\circ }$ at the critical incident angle $\xi_{1c}$, and it decreases after this maximum value. With reference to Figure 3, it means that the transmitted attenuation vector can never lie in the IV quadrant, but it is parallel to the interface when the incident angle is equal to its critical value. Finally, in Figure 9, the case $\varepsilon_{2}^{\prime \prime }=1.4>\varepsilon_{2c}^{\prime \prime }$ is considered. In this case, the transmitted attenuation angle cannot reach the value $90^{\circ }$, and it always remains less than such a value. With reference to Figure 3, it means that the transmitted attenuation vector always lies in the I quadrant.

## 4. Discussion

This article presented theoretical results concerning the electromagnetic penetration in lossy media by means of inhomogeneous plane waves. The physical properties of the wave responsible for deep penetration have been carefully investigated and the important result that only leaky waves of the improper type can be used allows for the possible design of suitable leaky-wave radiators.

A complete set of conditions for obtaining deep penetration has been analytically derived in terms of the physical properties of the involved media, thus permitting for defining the characteristics of the impinging inhomogeneous wave that optimize this promising phenomenon in different practical scenarios. A parametric analysis of the electromagnetic propagation in the lossy medium around the minimal condition that allows for deep penetration showed the limit of the phenomenon as well as the possibility to obtain even deeper effects, i.e., $\zeta_{2}>90^{\circ }$. This result can be justified, in a limited region, also in realistic scenarios in which a finite beam is considered. Let us consider a hypothetical LWA designed to meet the deep penetration condition for a specific material at the microwave frequencies; then, let us expose the LWA to a medium having same permittivity but lower conductivity: one would expect $\zeta_{2}>90^{\circ }$ because the second medium would offer less resistance to the electromagnetic radiation compared to the one for which the antenna was designed. Anyway, for certain media, an attempt to reach a larger value of the angle $\zeta_{2}$ may represent an objective difficult to meet because this could require a large value of the phase vector generated by the LWA, and, therefore, a challenging antenna design.

The objective of the described research is to build a solid theoretical support to practical applications. Results achieved in this paper will therefore be relevant to design practical radiating structures that can be used to reproduce the deep penetration scenario demonstrated here. An attempt to achieve the deep penetration condition ($\zeta_{2}\geq 90^{\circ }$) in a realistic application, i.e., in the case of a finite beam, will have to employ oblique incidence, as demonstrated here. An intuitive explanation can be pursued by analysing, again, Figure 4, and imagining a hypothetical observer who moves along the direction of power flow. Such an observer would not notice the evident increase of field that he would instead experience moving on different directions, such as the one marked with a red-dashed line in the figure: it follows that a separation surface orthogonal to the power flow would not allow deep penetration.

An exhaustive comparison in terms of penetration between leaky waves produced by finite sources and homogeneous waves is beyond the scope of this paper: anyway, a direct consequence of the results found here is that, when the deep penetration effect is obtained, a finite leaky wave guarantees penetration in the near field larger than the one obtained through homogeneous waves if the peak of the field amplitude at the interface with the lossy medium for the two waveforms coincides. This result has important implications in practical applications: employing an opportune leaky-wave antenna design, a requested amplitude of the electric field may be obtained in the lossy medium avoiding the boosting of the field at the interface, and reducing consequently the risks of overheating or burning of the surface layers that are present when more traditional homogeneous waves are employed. In other cases that may be of interest, e.g., when the peaks of the field amplitude for the two waveforms coincide in the absence of the lossy medium, but the homogeneous wave presents an amplitude of the field at the separation surface larger than the one of the leaky wave, the analysis of various factors, such as the shape of the beams, and the media considered, may be necessary for determining which waveform produces the larger penetration: in these cases, further investigations involving realistic antenna structures will have to be performed.

## 5. Conclusions

The current article presented an analytical study of the deep-penetration effect achievable by inhomogeneous plane waves. The impact of different materials on the deep-penetration condition were studied, and suitable finite waveforms, said leaky waves, were also indicated as a way to reach deep penetration in near field. Some of the limits and potentialities of those waveforms were also indicated. Future studies will focus on numerical simulations to determine the extent of this effect reachable by employing realistic and finite structures.
